# Supplementary material for: Towards enhanced functionality of vagus neuroprostheses through in silico optimized stimulation
Source: Nat Commun. 2024 Jul 20;15:6119. doi: 10.1038/s41467-024-50523-6 (PMC11271449; doi:10.1038/s41467-024-50523-6)
Supplement: Supplementary file 1 — Supplementary Information [file 41467_2024_50523_MOESM1_ESM.pdf]

SUPPLEMENTARY INFORMATION

Towards enhanced functionality of vagus neuroprostheses through in silico optimized stimulation

Federico Ciotti[1]†, Robert John[1]†, Natalija Katic Secerovic[1,2]†, Noemi Gozzi[1], Andrea Cimolato[1], Naveen Jayaprakash[3,4], Weiguo Song[3,4], Viktor Toth[3,4], Theodoros Zanos[3,4,5,6], Stavros Zanos[3,4,5,6], Stanisa Raspopovic[1]*

[1] Laboratory for Neuroengineering, Department of Health Sciences and Technology, Institute for Robotics and Intelligent Systems, ETH Zürich, Zürich, Switzerland.

[2] The Mihajlo Pupin Institute, University of Belgrade, Belgrade, Serbia

[3] Northwell Health, New Hyde Park, NY, USA

[4] Feinstein Institutes for Medical Research, Manhasset, NY, USA

[5] Donald and Barbara Zucker School of Medicine at Hofstra/Northwell, Hempstead, NY, USA

[6] Elmezzi Graduate School of Molecular Medicine, Manhasset, NY, USA

*Corresponding author: stanisa.raspopovic@hest.ethz.ch
† Equal contribution

List of Supplementary Materials:

- Supp. Figure 1. Convergence study for subsampling the fiber population.
- Supp. Figure 2. Distributions of fiber diameters and fiber locations of the sampled histological data used for M1 (left) and M2 (right) grouped by fiber type.
- Supp. Figure 3. Novel methodologies for optimizing computational efficiency – anodic stimulation.
- Supp. Figure 4. Sweeps of the time step dt for MRG (left) and TH (right) fibers.
- Supp. Figure 5. Fiber clustering.
- Supp. Figure 6. Additional results on model personalization and validation.
- Supp. Figure 7. Additional results regarding prediction of heart rate variation through personalized models
- Supp. Figure 8. Equivalent electrical circuits of myelinated and unmyelinated fibers for extracellular stimulation.
- Supp. Figure 9. Polynomial fits of the McIntyre-Richardson-Grill (MRG) model parameters.
- Supp. Table 1. Conductance of the materials applied in COMSOL.
- Supp. Table 2. Conductance values for the ion channels of unmyelinated fibers for the TH model [mS/cm2].
- Supp. Table 3. Coefficients of the polynomials fitted to the McIntyre-Richardson-Grill (MRG) model parameters.
- Supp. Table 4. Summary of the presented novel methodologies for the improvement of computational efficiency for all modeled fiber types.
- Supp. Listing 1. Algorithm for the dynamic discretization of unmyelinated fibers.
- Supp. Listing 2. Algorithm for the longitudinal truncation of myelinated and unmyelinated fibers.

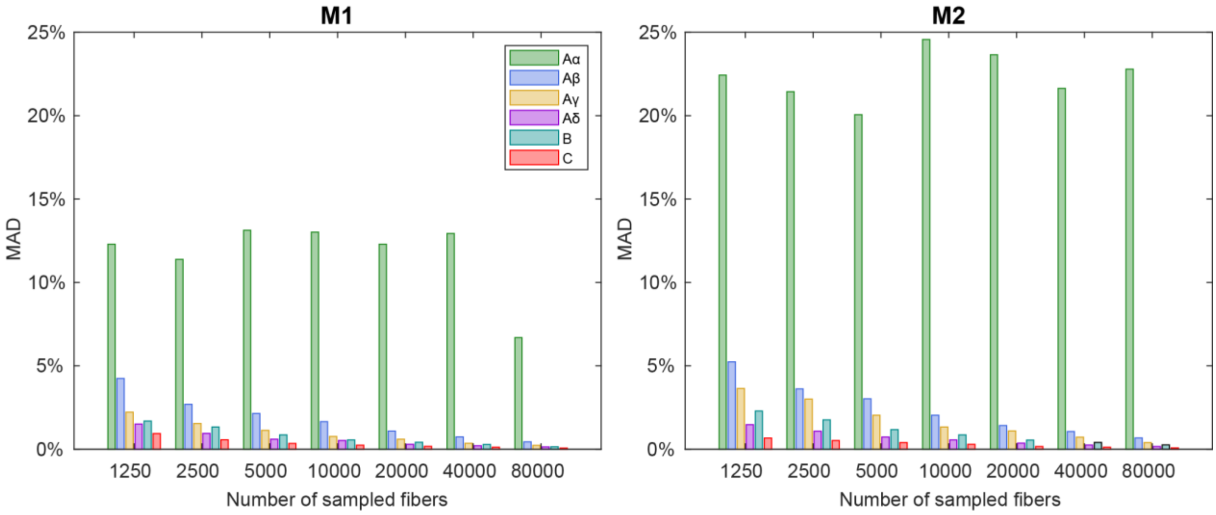

Supplementary Figure 1. Convergence study for subsampling the fiber population. To choose the number of fibers to sample, we computed the per-type recruitment curves from stimulating by each active site with a monopolar cathodic pulse of 500 µs pulse width models M1 and M2 with the complete fiber population. We then computed the mean absolute deviation between the recruitment curves computed with a randomly chosen subsample of a varying number of fibers between 1250 and 80000 fibers and the recruitment curves obtained with the complete population. It resulted in the choice of sampling about 10’000 fibers for subsequent studies, which limits the MAD under 2%, except for Aα fibers which we did not subsample due to their low occurrence.

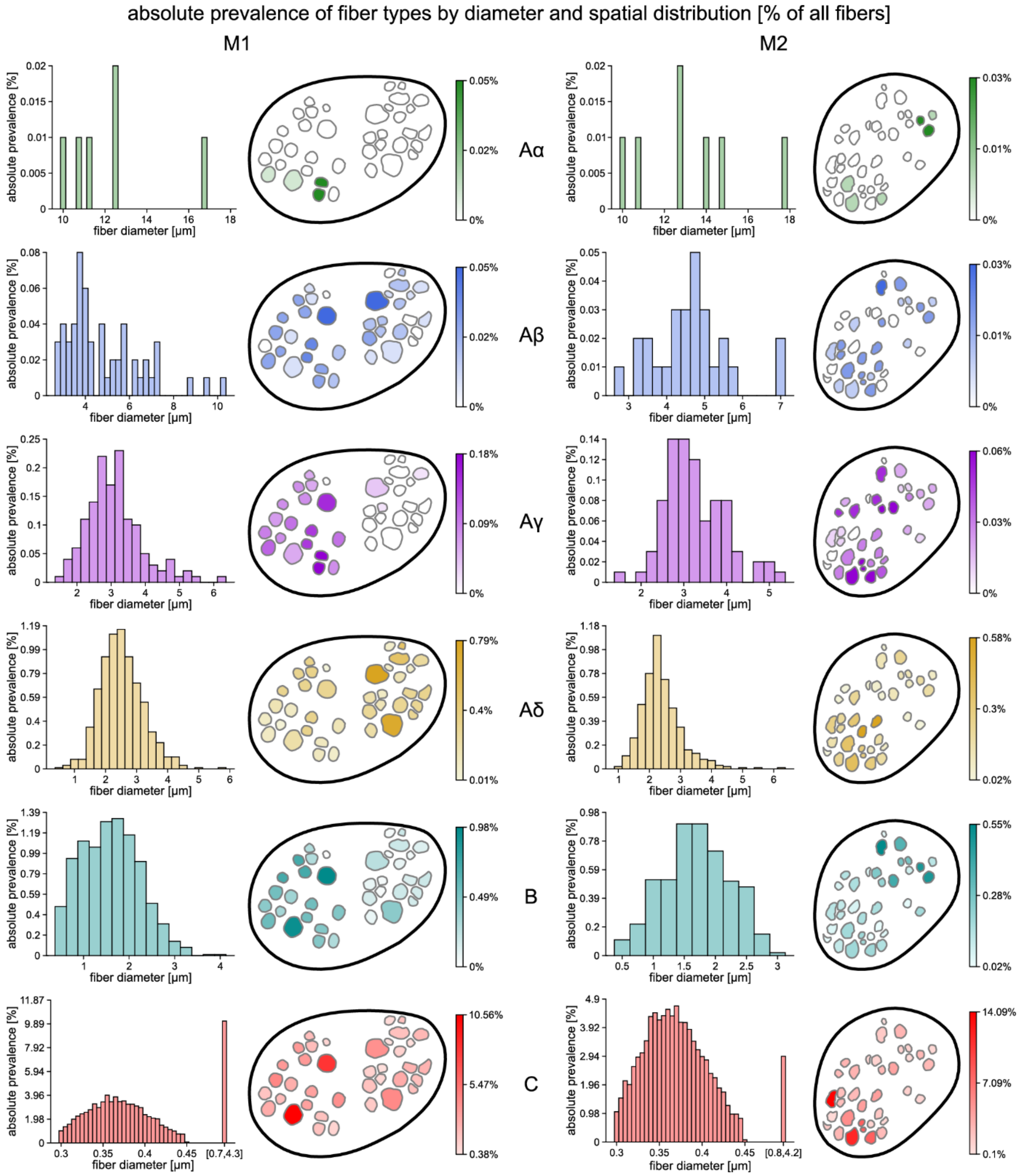

Supplementary Figure 2. Distributions of fiber diameters and fiber locations of the sampled histological data used for M1 (left) and M2 (right) grouped by fiber type. Values are given as a fraction of all fibers placed in the respective nerve model. Fiber diameters and locations were obtained from immunohistological analysis of cross section C2 (displayed).

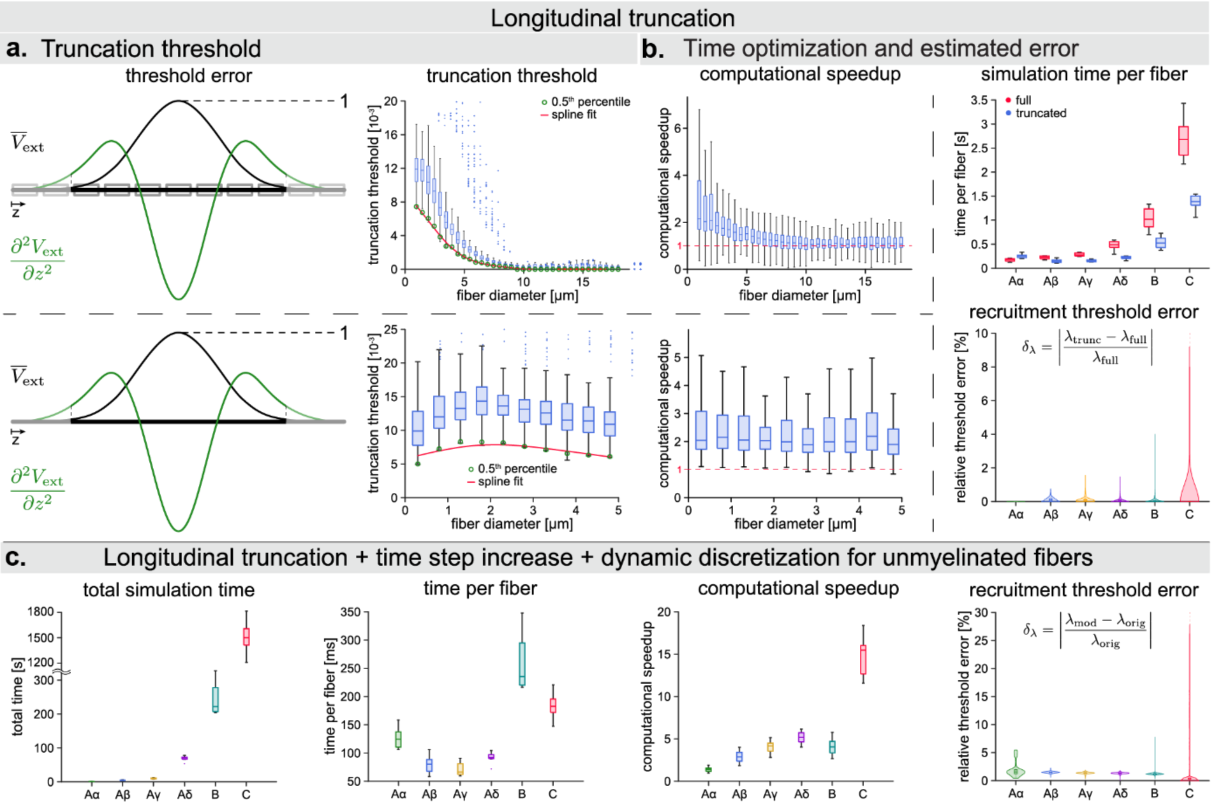

Supplementary Figure 3. **Novel methodologies for optimizing computational efficiency – anodic stimulation.** All studies were conducted by stimulating M1 with a monopolar anodic pulse of 500 µs pulse width. Outliers of boxplots are not displayed. (**a**) Illustration of longitudinal truncation for myelinated (top) and unmyelinated (bottom) fibers. The truncation threshold is chosen such that only sections relevant for predicting the physiological response, mediated by the positive part of the second spatial derivative of the extracellular potential, remain. Truncation thresholds determined by a study for myelinated (top) and unmyelinated (bottom) fibers ($n \approx 400$ per boxplot). (**b**) Computational speedup when using longitudinal truncation for myelinated (top) and unmyelinated (bottom) fibers ($n \approx 400$ per boxplot). Simulation time per fiber when using longitudinal truncation. Relative deviation of recruitment thresholds when using longitudinal truncation (n = Aα: 56; Aβ: 456; Aγ: 1104; Aδ: 5968; B: 7536; C: 65736). The relative deviations of recruitment thresholds are larger than 5% for 1732 (2.63%) of the C fiber data points, with the maximum relative error being 21.1%. Total simulation time per fiber type. Simulation time per fiber. (**c**) Computational speedup per fiber type when considering all presented methods. Relative deviation of recruitment thresholds when considering all presented methods while simulating with an accuracy of 0.1 (n = Aα: 56; Aβ: 456; Aγ: 1104; Aδ: 5968; B: 7536; C: 65736). The relative deviations of recruitment thresholds are larger than 5% for 1639 (2.49%) of the C fiber data points, with the maximum relative error being 107%.

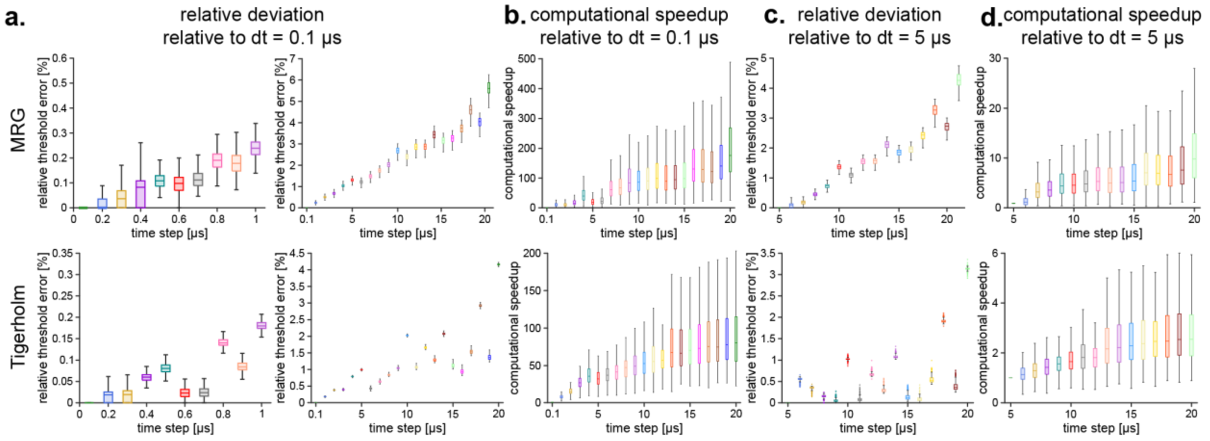

Supplementary Figure 4. **Sweeps of the time step dt for MRG (top) and TH (bottom) fibers.** Fiber diameters were assigned by uniformly sampling the discrete sets {1, 1.5, …, 18} μm (MRG fibers, n = 1694) and {0.3, 0.8, …, 4.8} μm (TH fibers, n = 478). All studies were conducted by stimulating using a monopolar cathodic pulse with 500 μs pulse width. Outliers of boxplots are not displayed. Note that NEURON by default employs the implicit Euler method, which is a convergent first order solver with global truncation error $O(\mathrm{dt})$[1]. (a) The recruitment thresholds deviations relative to the results obtained with dt = 0.1 μs, for which the results were deemed have sufficiently converged (deviation of recruitment thresholds from dt = 0.2 μs to dt = 0.1: 0.03 ± 0.04% for MRG; 0.02 ± 0.02% for TH). An approximately linear correlation can be observed, although there is significantly more jitter for TH than MRG fibers. For dt = 5 μs the recruitment thresholds deviate by 1.3 ± 0.1% for MRG and 1.0 ± 0.0% for TH fibers. (b) Computational speedup relative to simulations performed with dt = 0.1 μs. For dt = 5 μs the value is 23.0 ± 17.7 for MRG and 34.2 ± 14.2 for TH fibers. (c) Deviation of recruitment thresholds relative to dt = 5 μs, which is a standard value in literature for both models. The value for dt = 13 μs is 1.5 ± 0.1% for MRG and 0.3 ± 0.1% for TH fibers. (d) Speedup of simulations relative to those executed with dt = 5 μs. The value for dt = 13 μs is 6.6 ± 5.9 for MRG and 2.5 ± 1.3 for TH fibers.

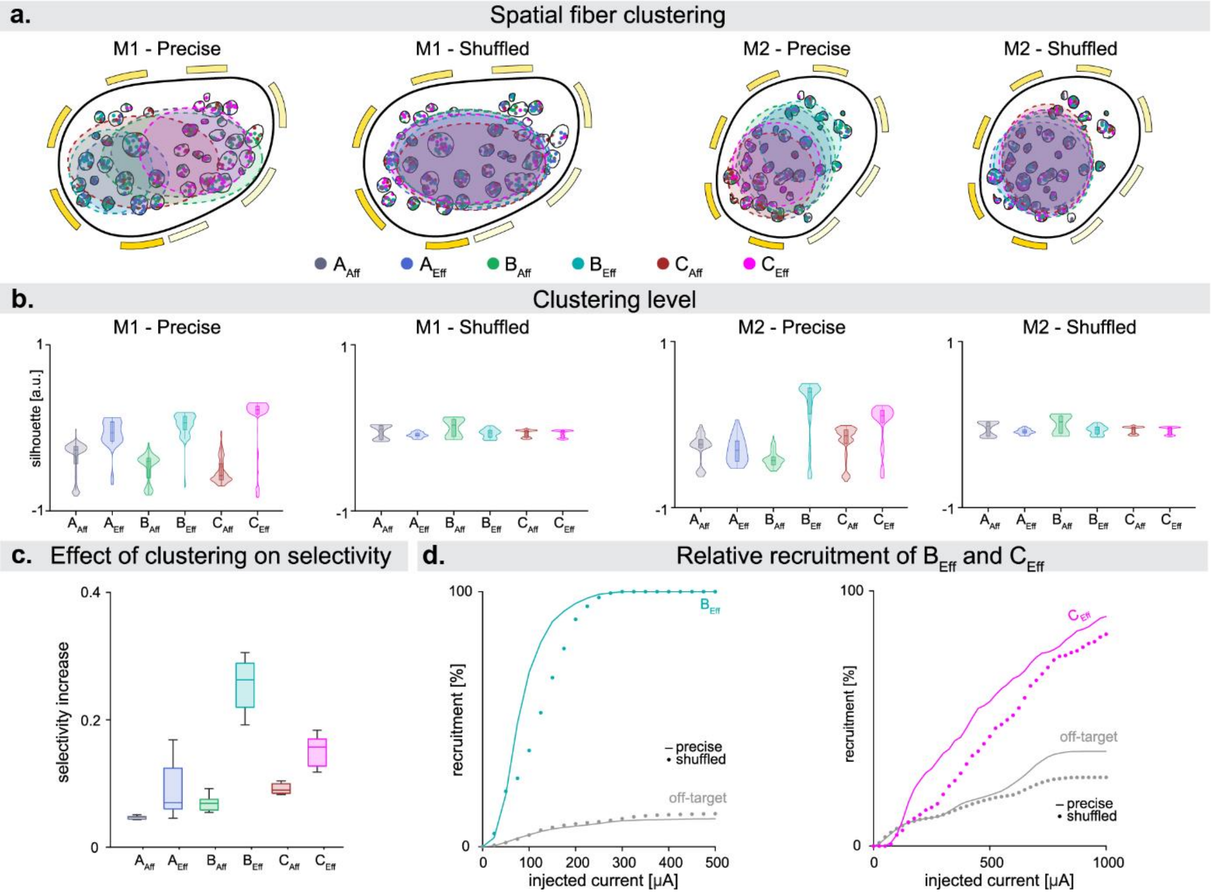

Supplementary Figure 5. Fiber clustering. (**a**) Cross section of the nerve models with the locations of randomly selected fibers, reported as colored circles (each group contains the same number of fibers to avoid over-representation of larger groups). The colored ellipses are centered on the center of mass of every fiber group and have semiaxes equal to ± 1.5 standard deviations of the fiber centers in both x and y. (**b**) Violin plots of the silhouette coefficient of each fiber group (n = 10107 for M1, 10208 for M2). (**c**) The maximum increase in selectivity for the most selective active site per fiber group (n = 8 per fiber type), within the stimulation range (0 – 1 mA). Nerve model M2 with histologically accurate fiber locations was compared to four shuffled models. (**d**) The recruitment curves for exemplary target fiber groups (B_{Eff} and C_{Eff}) for their most selective active sites are displayed for M2. Furthermore, the recruitment curves of off-target fiber groups are shown. These recruitment curves are plotted for both the model with histologically accurate fiber placements (solid lines) and the model with randomly shuffled fibers (dotted lines). They show the higher achievable target recruitment levels relative to the off-target recruitment thanks to the realistic fiber placement.

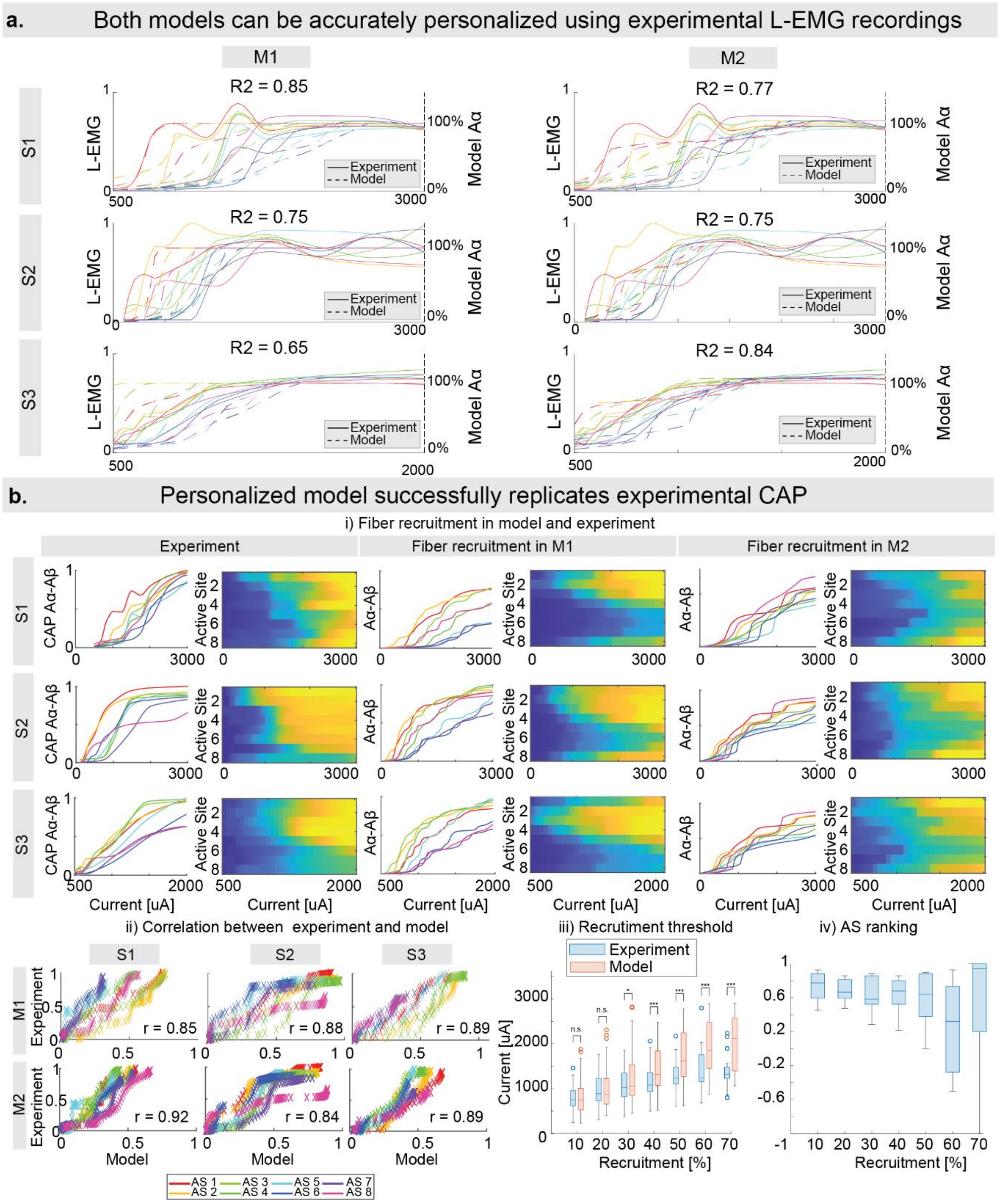

Supplementary Figure 6. Additional results on model personalization and validation. (**a**) Recruitment curves of M1 and M2 personalized to the three experimental subjects, overlaid to the experimentally measured L-EMG, for all active sites. (**b**) **i.** One row per subject, in the first and second column the normalized fast CAP curves are reported per active site are reported. In the third and fourth column, the corresponding recruitment curves of Aα and Aβ estimated by the personalized M1. In the fifth and sixth column, the same for M2. **ii.** The normalized CAP and M2 model-predicted recruitment level for each active site and current level are correlated on a scatter plot. Pearson's correlation coefficient is reported. **iii.** The distribution of thresholds to recruit 10% to 70% of Aα and Aβ fibers in the personalized models are compared to the experimentally measured thresholds to obtain a CAP value of 10% to 70% (two-sided Wilcoxon signed rank test, n = 48 per boxplot, p = 0.83, 0.36, 0.011, 0.0001, < 0.0001, 0.0002, 0.0001, respectively for increasing recruitment levels). **iv.** The ranking of active sites predicted by the personalized models is compared to the experiment by computing Spearman's correlation on thresholds, for recruitment levels between 10% and 70% (n = 42: 6 personalized models per boxplot).

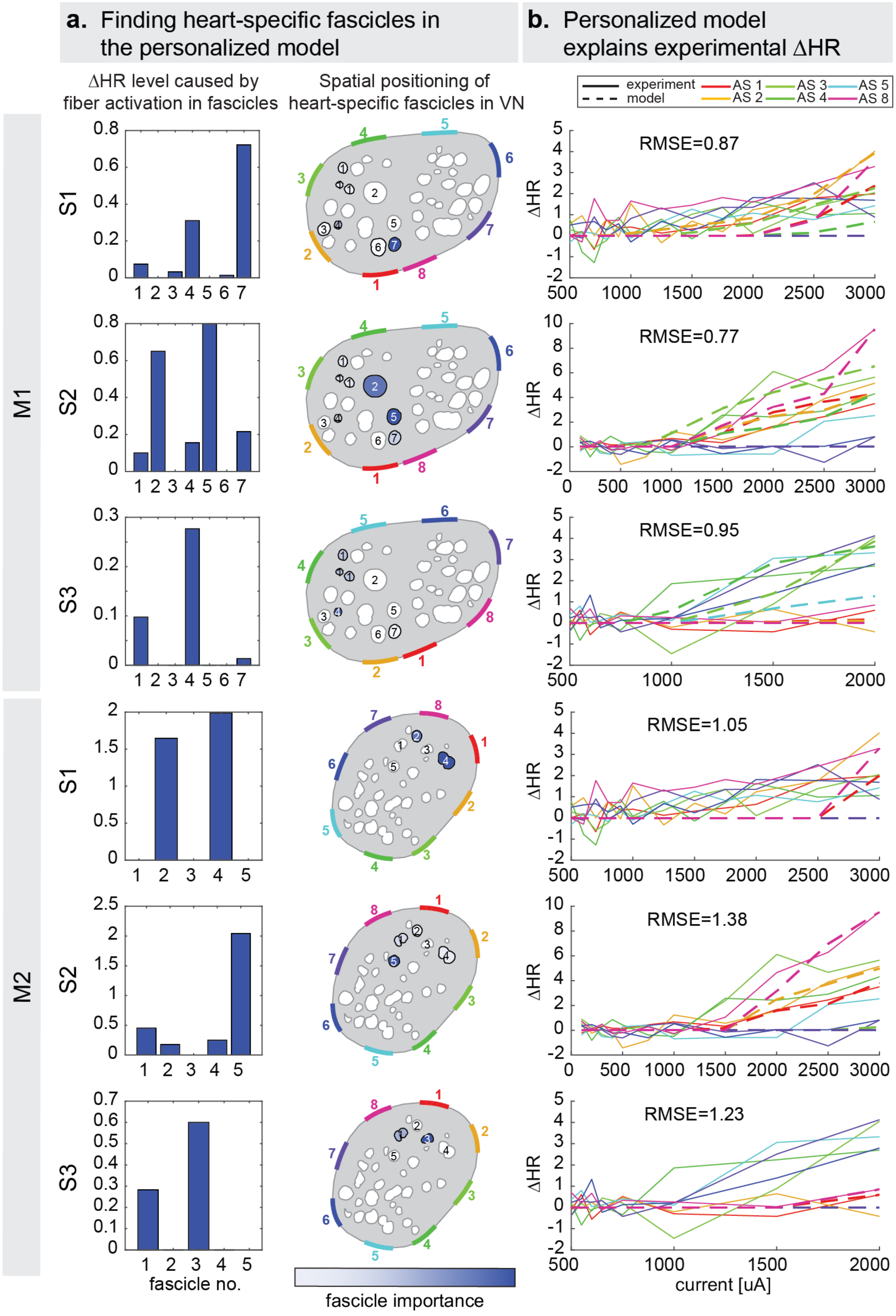

Supplementary Figure 7. Additional results regarding prediction of heart rate variation through personalized models **(a)** Finding heart-specific fascicles for both models personalized to the three experimental subjects. Level of HR change (left, bars) caused by fiber activation in specific fascicles. Fascicles are spatially presented and color-coded based on these values (right). **(b)** Recruitment curves of the models personalized to the three experimental subjects, overlaid to the experimentally measured ΔHR, for all active sites.

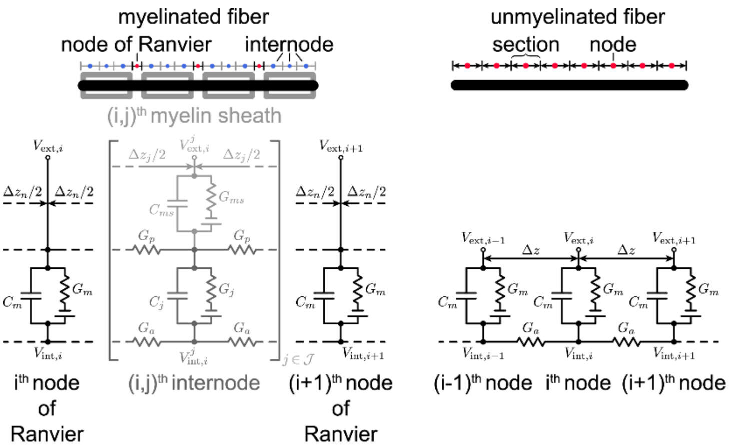

Supplementary Figure 8. Equivalent electrical circuits of myelinated and unmyelinated fibers for extracellular stimulation. The myelinated regions between nodes of Ranvier are usually discretized into multiple sections, which can be modeled by concatenating the displayed subcircuit. For instance, the MRG model is obtained by concatenating the subcircuits encoded by the set $\mathcal{J} = \{\mathrm{MYSA}, \mathrm{FLUT}, \mathrm{STIN}, \mathrm{STIN}, \mathrm{STIN}, \mathrm{STIN}, \mathrm{STIN}, \mathrm{STIN}, \mathrm{FLUT}, \mathrm{MYSA}\}$. Based on [2].

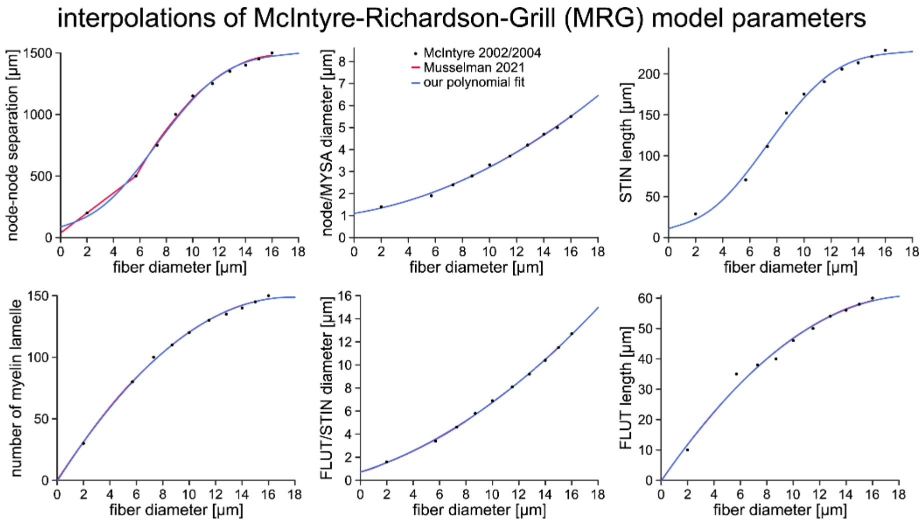

Supplementary Figure 9. **Polynomial fits of the McIntyre-Richardson-Grill (MRG) model parameters.** The data points were obtained from [3,4]. Note that the node-node separation was interpolated, but not actually used in the model, as it is an implicit result of assigning MYSA, FLUT, STIN and node lengths. Musselmann et al., 2021 conversely did not interpolate the STIN length. Node and MYSA length were respectively fixed at 1 µm and 3 µm.

Supplementary Table 1. Summary of the presented novel methodologies for the improvement of computational efficiency for all modeled fiber types. Evaluated by stimulating M1 with a monopolar cathodic pulse of 500 µs pulse width. All values are formatted as mean ± standard deviation.

fiber type	unit	Aα	Aβ	Aγ	Aδ	B	C
baseline							
total time	[s]	1.2±0.3	11.5±1.3	45.3±8.5	488.3±44.4	1591.2±428.9	23070.5±3201.8
time per fiber	[ms]	170.0±44.9	201.2±22.4	328.3±61.3	654.6±59.5	1689.2±455.4	2807.7±389.7
dynamic discretization							
total time	[s]	not applicable					2748.4±145.2
time per fiber	[ms]						334.5±17.7
computational speedup	[-]						8.1±1.4
relative threshold deviation	[%]						0.4±1.6
longitudinal truncation							
total time	[s]	1.0±0.2	3.0±0.4	7.1±1.3	60.2±3.8	365.4±33.3	1674.7±125.2
time per fiber	[ms]	140.0±34.8	52.5±6.6	51.5±9.2	80.7±5.1	387.8±35.4	1777.8±132.9
computational speedup	[-]	1.2±0.3	3.9±0.3	6.4±0.6	8.2±1.0	4.4±1.4	13.7±1.2
relative threshold deviation	[%]	0.0±0.0	0.2±0.3	0.1±0.2	0.0±0.2	0.0±0.2	0.3±1.6
time step increase							
total time	[s]	0.4±0.1	5.2±1.0	18.5±2.1	185.8±14.3	554.5±48.4	9631.3±2778.2
time per fiber	[ms]	62.5±11.4	91.8±17.3	134.1±15.1	249.1±19.2	588.6±51.4	1172.1±338.1
computational speedup	[-]	2.8±0.8	2.2±0.4	2.4±0.3	2.6±0.2	2.9±0.6	2.6±0.8
relative threshold deviation	[%]	1.6±0.8	1.6±0.8	1.5±0.2	1.4±0.2	1.2±0.2	0.3±0.1
longitudinal truncation + time step increase (+ dynamic discretization)							
total time	[s]	0.4±0.1	1.4±0.3	3.1±0.6	28.5±3.2	170.4±5.5	197.6±11.0
time per fiber	[ms]	63.2±12.1	25.2±5.1	22.7±4.0	38.3±4.2	180.9±5.9	24.0±1.3
computational speedup	[-]	2.8±0.8	8.2±1.3	14.5±1.5	17.3±2.5	9.3±2.1	117.3±18.7
relative threshold deviation	[%]	2.5±3.0	1.8±0.8	1.5±0.3	1.4±0.3	1.3±0.2	0.5±1.6

Supplementary Table 2. Conductance of the materials applied in COMSOL[5].

Material	Conductance [S/m]
epineurium	0.083
perineuria	0.00088
endoneuria (longitudinal)	0.571
endoneuria (transversal)	0.0826
saline	2
electrode substrate	10^{-13}

Supplementary Table 3. Conductance values for the ion channels of unmyelinated fibers for the TH model [mS/cm^2]. Listed are the values of the original publication [6], of the modelDB file published in [7] and used in [8] as well as the values we used in our implementation of the model. The utilized values are highlighted in bold. Note that the conductance values are very similar for almost all ion channels, with exception of pump and K_{Na}.

Channel	Tigerholm et al. 2014[6]	Pelot et al. 2020[7,8]	Ours
pump	0.0048	-4.7891	**0.0047891**
$Na_v1.7$	**106.6439**	106.64	**106.6439**
$Na_v1.8$	**242.7124**	242.71	**242.7124**
$Na_v1.9$	0.0948	**0.094779**	**0.094779**
K_{dr}	**18.0017**	18.002	**18.0017**
K_A	**12.7555**	12.756	**12.7555**
K_M	**6.9733**	6.9733	**6.9733**
h	**2.5377**	**2.5377**	**2.5377**
K_{Na}	**0.0012**	0.42	**0.0012**

Supplementary Table 4. Coefficients of the polynomials fitted to the McIntyre-Richardson-Grill (MRG) model parameters.

	x^6	x^5	x^4	x^3	x^2	x	1
node-node separation	-0.0003	0.0214	-0.4950	4.2415	-4.5773	38.6695	85.7439
number of myelin lamelle					-0.4749	16.8531	-0.7648
node/MYSA diameter					0.0109	0.1007	1.0988
FLUT/STIN diameter					0.0236	0.3673	0.7122
STIN length	-0.0001	0.0038	-0.0891	-0.7879	-1.1951	5.8044	10.8143
FLUT length					-0.1652	6.3538	-0.2862

Supplementary Table 5. Summary of the presented novel methodologies for the improvement of computational efficiency for all modeled fiber types. Evaluated by stimulating M1 with a monopolar anodic pulse of 500 µs pulse width. All values are formatted as mean ± standard deviation.

fiber type	unit	Aα	Aβ	Aγ	Aδ	B	C
**baseline**							
total time	[s]	1.2±0.2	12.7±1.9	39.0±4.8	356.6±70.4	972.3±217.7	22133.2±3403.6
time per fiber	[ms]	172.7±30.9	222.7±33.2	282.6±34.9	478.0±94.3	1032.2±231.1	2693.6±414.2
**dynamic discretization**							
total time	[s]	not applicable					not simulated (presumed to be similar to the results in Table 2)
time per fiber	[ms]						
computational speedup	[-]						
relative threshold deviation	[%]						
**longitudinal truncation**							
total time	[s]	1.7±0.4	8.5±2.1	21.5±3.2	169.6±40.0	500.6±116.2	11948.0±2659.9
time per fiber	[ms]	246.0±54.6	148.7±36.7	155.5±23.3	227.4±53.6	531.4±123.4	1454.1±323.7
computational speedup	[-]	0.7±0.2	1.5±0.2	1.9±0.4	2.2±0.7	0.5±0.1	1.9±0.2
relative threshold deviation	[%]	0.0±0.0	0.1±0.1	0.1±0.1	0.1±0.1	0.1±0.1	0.3±2.0
**time step increase**							
total time	[s]	not simulated (presumed to be similar to the results in Table 2)					
time per fiber	[ms]						
computational speedup	[-]						
relative threshold deviation	[%]						
**longitudinal truncation + time step increase (+ dynamic discretization)**							
total time	[s]	0.9±0.1	4.5±0.9	9.8±1.7	68.6±7.1	243.2±46.6	1507.9±181.1
time per fiber	[ms]	126.1±18.1	79.6±15.7	71.2±12.0	91.9±9.5	258.1±49.5	183.5±22.0
computational speedup	[-]	1.4±0.3	2.9±0.7	4.1±0.7	5.2±0.7	4.1±1.1	14.8±2.3
relative threshold deviation	[%]	2.0±1.3	1.5±0.2	1.4±0.2	1.3±0.1	1.2±0.2	0.7±2.0

Supplementary Listing 1. **Algorithm for the dynamic discretization of unmyelinated fibers.** Dynamic discretization of a single fiber exposed to static (multipolar) stimulation is presented. Lists are marked in bold and the index 0 is assigned to the leading entry. The algorithm requires a list $\mathbf{V}_{\text{ext}} \in \mathbb{R}^{1\times n_{\text{nodes}}}$ containing the values of the superimposed extracellular potential at each initial fiber node, the locations of which were obtained by fixed discretization using section length dz_{init}. Furthermore, a minimum and maximum section length dz_{min} and dz_{max} for the dynamically discretized fiber need to be defined. Dynamic discretization estimates the second spatial derivative of the extracellular potential, which is normalized and then used to assign section lengths to each node by linearly interpolating between dz_{min} and dz_{max}. Sections are then iteratively concatenated, and the extracellular potential is linearly interpolated at the new nodes. Returned is an ordered list $\widehat{\mathbf{dz}} \in \mathbb{R}^{1\times \hat{n}_{\text{nodes}}}$ of section lengths as well as a list $\hat{\mathbf{V}}_{\text{ext}} \in \mathbb{R}^{1\times \hat{n}_{\text{nodes}}}$ containing the values of the interpolated extracellular potential.

Algorithm 1: dynamic discretization of unmyelinated fibers

```
Input: V_ext ∈ ℝ^(1×n_nodes), dz_init, dz_max, dz_min,
n_nodes ← number of entries in V_ext
n_sections ← n_nodes − 1
l_fiber ← n_sections · dz_init
ẑ_nodes ← list with a single 0 entry
dz ← empty list with n_nodes entries
dẑ ← empty list

Tangentially extrapolate V_ext by 5% of the total fiber length at both ends
V''_ext ← central differences applied twice to extrapolated V_ext
Smooth V''_ext with 2nd order forward-backward Butterworth low pass SOS filter
Remove extrapolated data from smoothed V''_ext
V''_ext,+ ← V''_ext with negative values set to 0
V''_ext,− ← V''_ext with positive values set to 0
V̄''_ext,+ ← normalized V''_ext,+
V̄''_ext,− ← normalized V''_ext,−
V̄''_ext ← V̄''_ext,+ + V̄''_ext,−
for i ← 0 to n_nodes − 1 do
    if i ≠ n_nodes − 1 then
        dz_i ← dz_min + (dz_max − dz_min) (1 − (V̄''_ext,i + V̄''_ext,i+1) / 2)
    else
        dz_i ← dz_min + (dz_max − dz_min) (1 − V̄''_ext,i)
    end
end
i ← 0
while ẑ_nodes,i < l_fiber do
    Determine the node j closest to ẑ_nodes,i along the fiber
    dz_iter,max ← l_fiber − ẑ_nodes,i
    if dz_j ≤ dz_iter,max then
        Append dz_j to dẑ
    else
        Append dz_iter,max to dẑ
    end
    Append sum of all entries in dẑ to ẑ_nodes
    i ← i + 1
end
V̂_ext ← V_ext linearly interpolated at all entries in ẑ_nodes
return: dẑ ∈ ℝ^(1×n̂_nodes), V̂_ext ∈ ℝ^(1×n̂_nodes)
```

Supplementary Listing 2. Algorithm for the longitudinal truncation of myelinated and unmyelinated fibers (pseudocode). Longitudinal truncation of a single fiber exposed to static (multipolar) stimulation is presented. Lists and arrays are marked in bold and the index 0 is assigned to the leading entry. The algorithm requires a list $\mathbf{V}_{\text{ext}} \in \mathbb{R}^{\mathbf{1}\times \boldsymbol{n}_{\mathbf{nodes}}}$, containing the values of the superimposed extracellular potential at each fiber node, as well as the upper bound for the recruitment threshold value λ_{max}, its estimation precision λ_{prec} and the permitted relative deviation during truncation $\lambda_{\text{tol,init}}$. The variable modelType $\in$ {'myelinated', 'unmyelinated'} indicates whether the fiber is myelinated or unmyelinated. Longitudinal truncation applies a bisection method to the extracellular potential to determine a threshold value of the same, which then indicates adequate locations for the truncation of a fiber. Returned are the truncation indices node_{lb} and node_{ub}, as well as a list $\hat{\mathbf{V}}_{\text{ext}} \in \mathbb{R}^{\mathbf{1}\times \hat{\boldsymbol{n}}_{\mathbf{nodes}}}$ containing the values of the extracellular potential truncated at these nodes. Additionally, the truncation threshold $V_{\text{ext,threshold}}$ is determined. Note that in the actual implementation the truncation nodes are not explicitly calculated for each fiber (as this would defeat the purpose of this method regarding the decrease of simulation time). Instead, a study is conducted *a-priori* to obtain a diameter dependent spline fit for $V_{\text{ext,threshold}}$, which is then sampled and evaluated on the relevant extracellular potential to establish the truncation nodes.

Algorithm 2: longitudinal truncation of myelinated and unmyelinated fibers

```
Input: V_ext ∈ ℝ^{1×n_nodes}, λ_max, λ_prec, λ_tol,init, modelType
node_lb ← 0
node_ub ← (number of entries in V_ext) − 1
node_lb,prev ← −1
node_ub,prev ← −1
λ_thresh,iter ← ∞

λ_thresh,init ← findThreshold(λ_max, λ_prec, V_ext)
if λ_thresh,init is not NaN then
    λ_tol ← max(λ_prec, λ_thresh,init · λ_tol,init)
    V_ext,lb ← 0
    V_ext,ub ← max(|V_ext,init|)
    while node_lb ≠ node_lb,prev & node_ub ≠ node_ub,prev & |λ_thresh,iter − λ_thresh,init| > λ_tol
     do
        V_ext,iter ← entries node_lb to node_ub of V_ext
        λ_thresh,iter ← findThreshold(λ_max, λ_prec, V_ext,iter)
        V_ext,truncation ← V_ext,lb + ((V_ext,ub − V_ext,lb) / 2)
        if |λ_thresh,iter − λ_thresh,init| ≤ λ_tol then
            V_ext,lb ← V_ext,truncation
        else
            V_ext,ub ← V_ext,truncation
        end
        ε_{V_ext} ← sorted indices of all entries of |V_ext,init| that are larger than V_ext,truncation
        node_lb,prev ← node_lb
        node_ub,prev ← node_ub
        if modelType is 'myelinated' then
            nodeRanvier_lb ← ⌊min(ε_{V_ext})/11 − 1⌋ + 1
            nodeRanvier_ub ← ⌈max(ε_{V_ext})/11⌉
            node_lb ← 11 · nodeRanvier_lb
            node_ub ← 11 · nodeRanvier_ub
        else if modelType is 'unmyelinated' then
            node_lb ← min(ε_{V_ext})
            node_ub ← max(ε_{V_ext})
        end
    end
    V̂_ext ← V_ext,iter
    V_ext,threshold ← V_ext,truncation / max(|V_ext,init|)
else
    node_lb ← NaN
    node_ub ← NaN
    V̂_ext ← NaN
    V_ext,threshold ← NaN
end
return: node_lb, node_ub, V̂_ext ∈ ℝ^{1×n̂_nodes}, V_ext,threshold
```

Supplementary Materials References:

1. Kavetski, D., Binning, P. & Sloan, S. W. Adaptive backward Euler time stepping with truncation error control for numerical modelling of unsaturated fluid flow. *International Journal for Numerical Methods in Engineering* **53**, 1301–1322 (2002).
2. Rattay, F. Analysis of Models for External Stimulation of Axons. *IEEE Transactions on Biomedical Engineering* **BME-33**, 974–977 (1986).
3. McIntyre, C. C., Richardson, A. G. & Grill, W. M. Modeling the excitability of mammalian nerve fibers: influence of afterpotentials on the recovery cycle. *J. Neurophysiol.* **87**, 995–1006 (2002).
4. McIntyre, C. C., Grill, W. M., Sherman, D. L. & Thakor, N. V. Cellular Effects of Deep Brain Stimulation: Model-Based Analysis of Activation and Inhibition. *Journal of Neurophysiology* **91**, 1457–1469 (2004).
5. Raspopovic, S., Petrini, F. M., Zelechowski, M. & Valle, G. Framework for the Development of Neuroprostheses: From Basic Understanding by Sciatic and Median Nerves Models to Bionic Legs and Hands. *Proceedings of the IEEE* (2017) doi:10.1109/JPROC.2016.2600560.
6. Tigerholm, J. *et al.* Modeling activity-dependent changes of axonal spike conduction in primary afferent C-nociceptors. *Journal of Neurophysiology* **111**, 1721–1735 (2014).
7. Pelot, N. A. *et al.* Excitation properties of computational models of unmyelinated peripheral axons. *Journal of Neurophysiology* **125**, 86–104 (2020).
8. Musselman, E. D., Cariello, J. E., Grill, W. M. & Pelot, N. A. ASCENT (Automated Simulations to Characterize Electrical Nerve Thresholds): A pipeline for sample-specific computational modeling of electrical stimulation of peripheral nerves. *PLOS Computational Biology* **17**, e1009285 (2021).
