# Supplementary material for: Towards enhanced functionality of vagus neuroprostheses through in silico optimized stimulation
Source: Nat Commun. 2024 Jul 20;15:6119. doi: 10.1038/s41467-024-50523-6 (PMC11271449; doi:10.1038/s41467-024-50523-6)
Supplement: Supplementary file 3 — Reporting Summary [file 41467_2024_50523_MOESM3_ESM.pdf]

nature portfolio

Corresponding author(s): Stanisa Raspopovic

Last updated by author(s): Jun 19, 2024

Reporting Summary

Nature Portfolio wishes to improve the reproducibility of the work that we publish. This form provides structure for consistency and transparency in reporting. For further information on Nature Portfolio policies, see our Editorial Policies and the Editorial Policy Checklist.

Statistics

For all statistical analyses, confirm that the following items are present in the figure legend, table legend, main text, or Methods section.

n/a	Confirmed	
☐	☒	The exact sample size (*n*) for each experimental group/condition, given as a discrete number and unit of measurement
☐	☒	A statement on whether measurements were taken from distinct samples or whether the same sample was measured repeatedly
☐	☒	The statistical test(s) used AND whether they are one- or two-sided<br>*Only common tests should be described solely by name; describe more complex techniques in the Methods section.*
☐	☒	A description of all covariates tested
☐	☒	A description of any assumptions or corrections, such as tests of normality and adjustment for multiple comparisons
☐	☒	A full description of the statistical parameters including central tendency (e.g. means) or other basic estimates (e.g. regression coefficient) AND variation (e.g. standard deviation) or associated estimates of uncertainty (e.g. confidence intervals)
☐	☒	For null hypothesis testing, the test statistic (e.g. *F*, *t*, *r*) with confidence intervals, effect sizes, degrees of freedom and *P* value noted<br>*Give P values as exact values whenever suitable.*
☒	☐	For Bayesian analysis, information on the choice of priors and Markov chain Monte Carlo settings
☒	☐	For hierarchical and complex designs, identification of the appropriate level for tests and full reporting of outcomes
☐	☒	Estimates of effect sizes (e.g. Cohen's *d*, Pearson's *r*), indicating how they were calculated

Our web collection on statistics for biologists contains articles on many of the points above.

Software and code

Policy information about availability of computer code

Data collection	All physiological data was collected using LabChart v8 (ADInstruments Inc).
Data analysis	The current iteration of the pipeline employs MATLAB 2022a, Python 3.9.12, COMSOL Multiphysics 5.6, and NEURON 8.2.1. Histological slices were segmented with ImageJ 1.52p and NeuronJ 1.4.3. The 3D models were created using Solidworks 2020 SP04. All original code, including modeling pipeline and online platform, was deposited on Zenodo and publicly accessible at https://doi.org/10.5281/zenodo.11219384.

For manuscripts utilizing custom algorithms or software that are central to the research but not yet described in published literature, software must be made available to editors and reviewers. We strongly encourage code deposition in a community repository (e.g. GitHub). See the Nature Portfolio guidelines for submitting code & software for further information.

Data

Policy information about availability of data

All manuscripts must include a data availability statement. This statement should provide the following information, where applicable:

- Accession codes, unique identifiers, or web links for publicly available datasets
- A description of any restrictions on data availability
- For clinical datasets or third party data, please ensure that the statement adheres to our policy

All data supporting the findings of this study are available within the article and its supplementary files. Any additional requests for information can be directed to, and will be fulfilled by, the corresponding authors. Source data are provided with this paper. The data required for the visualization of results on the online platform

was deposited on Zenodo65

and publicly accessible at https://zenodo.org/records/11219384.

Research involving human participants, their data, or biological material

Policy information about studies with human participants or human data. See also policy information about sex, gender (identity/presentation), and sexual orientation and race, ethnicity and racism.

Reporting on sex and gender	N/A
Reporting on race, ethnicity, or other socially relevant groupings	N/A
Population characteristics	N/A
Recruitment	N/A
Ethics oversight	N/A

Note that full information on the approval of the study protocol must also be provided in the manuscript.

Field-specific reporting

Please select the one below that is the best fit for your research. If you are not sure, read the appropriate sections before making your selection.

☒ Life sciences ☐ Behavioural & social sciences ☐ Ecological, evolutionary & environmental sciences

For a reference copy of the document with all sections, see nature.com/documents/nr-reporting-summary-flat.pdf

Life sciences study design

All studies must disclose on these points even when the disclosure is negative.

Sample size	The intra-subject results were obtained on models each composed by ~10'000 fibers, sample size chosen by a convergence study from the complete fiber population of more than 100'000 fibers. Initial evidence on inter-subject repeatability of the results was obtained by independently developing two realistic models. Each of these models was then personalized to three subjects (a total of 6 personalized models). No statistical methods were used to predetermine sample size for inter-subject observations due to the exploratory nature of the study.
Data exclusions	No data were excluded.
Replication	All information required for reproducibility are provided. The results were independently reproduced by models produced on two different experimental subjects, each applied on three previously unseen subjects. No attempts of replication were unsuccessful.
Randomization	Randomization is not relevant since no experimental groups were defined.
Blinding	Blinding is not relevant since no experimental groups were defined.

Reporting for specific materials, systems and methods

We require information from authors about some types of materials, experimental systems and methods used in many studies. Here, indicate whether each material, system or method listed is relevant to your study. If you are not sure if a list item applies to your research, read the appropriate section before selecting a response.

Materials & experimental systems

n/a	Involved in the study	
☒	☐	Antibodies
☒	☐	Eukaryotic cell lines
☒	☐	Palaeontology and archaeology
☐	☒	Animals and other organisms
☒	☐	Clinical data
☒	☐	Dual use research of concern
☒	☐	Plants

Methods

n/a	Involved in the study	
☒	☐	ChIP-seq
☒	☐	Flow cytometry
☒	☐	MRI-based neuroimaging

Animals and other research organisms

Policy information about studies involving animals; ARRIVE guidelines recommended for reporting animal research, and Sex and Gender in Research

Laboratory animals	Experiments were carried out on 5 Yucatan pigs of around 1 year of age and 40 kg weight, from Premier BioSource. Two of these were used to develop the models, three for the physiological experiments.
Wild animals	N/A
Reporting on sex	All animals were male. Sex-based analysis were not performed and sex was not considered during study design.
Field-collected samples	N/A
Ethics oversight	All procedures were performed in accordance with protocols approved by the Animal Care and Use Committee of Feinstein Institutes for Medical Research and New York Medical College.

Note that full information on the approval of the study protocol must also be provided in the manuscript.

Plants

Seed stocks	N/A
Novel plant genotypes	N/A
Authentication	N/A
